# Asystolic cardiac arrest following liposomal amphotericin B infusion: anaphylaxis or compliment activation-related pseudoallergy?

**DOI:** 10.1186/s13223-021-00582-x

**Published:** 2021-07-29

**Authors:** George P. Drewett, Ana Copaescu, Joseph DeLuca, Natasha E. Holmes, Jason A. Trubiano

**Affiliations:** 1 Department of Infectious Diseases, Austin Health, Heidelberg, Australia; 2 Department of Drug and Antibiotic Allergy Services, Austin Health, Heidelberg, Australia; 3 Department of Clinical Immunology and Allergy, McGill University Health Centre, Montréal, Canada; 4 Department of Medicine (Austin Health), University of Melbourne, Heidelberg, Australia; 5 Data Analytics Research and Evaluation (DARE) Centre, Austin Health and University of Melbourne, Heidelberg, Australia

**Keywords:** Ambisome, LAmB, Skin prick testing, Intradermal testing, CARPA

## Abstract

Allergic reaction to liposomal amphotericin B is rare. We report a case of cardiac arrest in a 64-year-old woman following liposomal amphotericin B infusion, requiring resuscitation. We also present the results of subsequent skin prick and intradermal testing to liposomal amphotericin on the patient and three healthy controls, highlighting the need for further research into the immunopathogenesis of this reaction.

## Background

Despite its widespread use, reports of severe reactions to liposomal amphotericin B (AmBisome^®^; LAmB), including anaphylaxis, are uncommon. Here we report a case of possible anaphylaxis or compliment activation-related pseudoallergy (CARPA) with asystolic cardiac arrest in an adult woman shortly after delivery of LAmB.

## Case presentation

A 64-year-old patient presented with a 2-week history of headache, mild photophobia, vomiting and confusion, with drenching night sweats but no fever or nuchal rigidity, and a diffuse rash involving the palms and soles. Initial investigations suggested both neurosyphilis [serum rapid plasma reagin (RPR) 1:256; *Treponema pallidum* particle agglutination assay (TPPA) reactive] and potentially cryptococcal meningitis (serum cryptococcal antigen low positive titre of 1:2). A CT performed on admission was unremarkable. Her past medical history was significant for hypertension, atrial fibrillation and depression, managed with Candesartan HCT 32/12.5 mg daily (withheld during admission), Sotalol 80 mg BD, Apixaban 5 mg BD (withheld during admission), and dothiepin 75 mg daily, with no known drug allergy or atopy history. A lumbar puncture was performed and the patient commenced on intravenous benzylpenicillin 4-h for treatment of neurosyphilis, LAmB 4 mg/kg daily and flucytosine 25 mg/kg 6-h as per Australian guidelines for management of cryptococcal meningitis [1].

The patient was normotensive (133/65 mmHg) at the time of commencing the infusion. Five minutes after completion of the initial LAmB infusion (total 320 mg infused over 1 h), she reported feeling light-headed and unwell, with ascending paraesthesia in her lower limbs. An electrocardiogram was performed, demonstrating a junctional bradycardia with a heart rate of 20 bpm. Immediately thereafter, the patient lost cardiac output and cardiac monitoring demonstrated asystolic arrest. No rash or bronchospasm were noted. The patient was normoglycaemic, serum potassium measured at this time was 4.3 mmol/L; serum lactate was 9.9 mmol/L. Cardiopulmonary resuscitation (CPR) was performed for 15 min and two sequential doses of 1 mg intravenous adrenaline were delivered. Return of spontaneous circulation was achieved, the patient was intubated, and transferred to the intensive care unit for ongoing monitoring and care. High sensitivity troponin I taken 4 h after the event was mildly elevated (36 ng/L, Reference range < 10 ng/L), likely due to the brief period of asystole and CPR. The following morning, the patient awoke without evidence of neurological deficit and was successfully extubated.

Serial tryptase measurements taken 1 and 6 h after the event were 11.2 mcg/L and 6.5 mcg/L (Ref range < 11mcg/L), and subsequent baseline tryptase taken 6 days after the event was 6.6 mcg/L, consistent with a mast-cell mediated hypersensitivity reaction based on the “20% + 2 rule” [2–4]. Specific IgE for latex and chlorhexidine were negative. Subsequent transthoracic echocardiogram, myocardial perfusion imaging, and baseline ECG were normal. Lumbar puncture demonstrated a negative cerebrospinal fluid cryptococcal antigen. As such, antifungal therapy was not recommenced as the serum cryptococcal antigen was considered a likely false positive. The patient was discharged to complete a 2-week course of intravenous benzylpenicillin for neurosyphilis with clinical resolution of symptoms. At outpatient follow-up 1 month later, subsequent skin-prick testing (SPT) of LAmB at 1:10 (4 mg/mL) and intradermal testing (IDT) at 1:1000 (0.04 mg/ml) and 1:100 (0.4 mg/ml) dilutions were negative. Our patient refused SPT and IDT to higher concentrations of LAmB secondary to concerns about possible reaction. IDT was also performed on three healthy controls at 1:100 concentration, without reaction.

## Discussion and conclusions

Amphotericin B is a polyene antifungal agent with a broad spectrum of activity. It is indicated for the treatment of serious fungal infections, including invasive disease and cryptococcal meningitis, and infusion reactions are not uncommon [5]. LAmB is a lipid formulation of amphotericin B that alters its pharmacokinetics, and is associated with fewer nephrotoxic and infusion-related adverse effects than conventional amphotericin B (amphotericin B desoxycholate) [6].

The liposome creates a spherical vesicle around the Amphotericin B molecules, changing its pharmacokinetics to reduce toxicity by facilitating targeted administration of the Amphotericin B by binding to the fungal cell walls, while at the same time protecting human cells from exposure to Amphotericin B [7]. The excipients contained in LAmB formulations are hydrogenated soy phosphatidylcholine, distearoylphosphatidylglycerol and cholesterol (which form the liposome), in addition to alpha tocopherol, sucrose, disodium succinate hexahydrate, sodium hydroxide and hydrochloric acid (as buffering agents).

Severe reactions to LAmB, including anaphylaxis, are uncommon. Potential cardiac toxicity (transient asystole) associated with amphotericin B was first reported in 1983 [8]. Since then, a review of the literature has identified nine reports of anaphylaxis or cardiac toxicity attributed to LAmB (Table 1). Of note, all the cases of cardiac toxicity appeared to be dose and time dependent (occurring only after multiple sequential doses) and were associated with hyperkalaemia [8–11], for which amphotericin B has been assigned a “black box warning,” however our case was normokalaemic at the time of event. Three of the reported cases of LAmB anaphylaxis were administered conventional amphotericin B either prior to or following LAmB without reaction [12–14], suggesting allergic reaction to the liposome or other excipients.

**Table 1:** Previously published cases of anaphylaxis and cardiac toxicity to LAmB

Author (journal)	Date published	Description	Comments
DeMonaco et al. (Drug Intelligence Clinical Pharmacology)[8]^a^	1983	76-year-old semi-comatose patient with renal failure who experienced two episodes of transient asystole associated with hyperkalaemia and supratherapeutic digoxin levels, temporally associated with amphotericin B infusion	First reported case of possible clinical cardiac toxicity
Laing et al. (Lancet) [12]	1994	29-year-old AIDS patient developed hypotension, erythema, fever, bronchospasm and facial oedema shortly after commencing LAmB infusion. Recovered after administration of IV adrenaline and hydrocortisone. No reaction to subsequent therapy with conventional amphotericin B	Likely reaction to liposomal component or excipient, given patient tolerated intravenous conventional amphotericin B after the reaction
Torre et al. (Annals of Pharmacotherapy)[16]	1996	10-year-old with Crohn’s disease, treated with LAmB for candidaemia. On Day 4 of treatment, developed diffuse erythema. On D5, developed flushing, erythema, hypertension, bradycardia and bronchospasm, treated with IV methylprednisolone. Not rechallenged	The delayed time course is atypical for IgE mediated anaphylaxis in this case
Schneider et al. (British Journal of Haematology)[13]	1998	40-year-old with haematological malignancy. Previously tolerated oral amphotericin B without reaction. Developed hypotension, asystole and bronchospasm shortly after LAmB infusion. Death 36 h later secondary to resultant cerebral oedema	Likely reaction to liposome component or excipient, given pt had tolerated oral amphotericin B formulation previously
el-Dawlatly et al. (Middle East Journal of Anaesthesiology)[11]	1999	39-year-old with systole during amphotericin B infusion for systemic aspergillosis. Associated with hyperkalaemia	Possible anaphylaxis, although acute hyperkalaemia is alternative explanation
Vaida et al. (Annals of Pharmacotherapy)[14]	2002	2-year-old with haematological malignancy. Tolerated intravenous amphotericin B deoxycholate without reaction. Changed to LAmB to manage supplementary potassium requirements. Shortly after commencing infusion, child became agitated, then developed erythema and facial swelling. Treated with oxygen and IV hydrocortisone, with complete resolution. Tolerated oral amphotericin B deoxycholate subsequently without complication	Likely reaction to liposomal component or excipient, given patient tolerated intravenous and oral formulations of conventional amphotericin B before and after the reaction
Groot et al. (Netherlands Journal of Medicine)[9]	2008	36-year-old received multiple doses of LAmB, 4 of which were associated with hyperkalaemia, the last of which resulted in fatal cardiac arrest	Appears to be a progressive dose- and time-related reaction
Kholve et al. (Journal of Antimicrobial Chemotherapy)[17]	2009	2 reported anaphylactic reactions in case series of 84 children receiving prophylactic or therapeutic LAmB in the context of malignancy/haematopoietic stem cell transplant	No detailed information about these reactions provided
Anonymous [Reactions Weekly (Aukland)] (not peer reviewed) [18]	2012	38-year-old with haematological malignancy commenced on LAmB, developed chest pressure, hypotension, hypoxia and dyspnoea shortly after commencing infusion. Resolved with IV fluid, steroids and diphenhydramine. Successful administration of LAmB following “desensitisation” by administering full dose over 11 h	Possible anaphylactic reaction although no reaction following subsequent administration raises some doubt
Sanches et al. (BMJ Case Reports) [10]	2014	9-month old developed atrioventricular blockade following 3 days of LAmB therapy, resolving on sessation of LAmB	Appears to be a progressive dose- and time-related reaction

^a^The first article in this table describes a reaction to conventional amphotericin B and is included as a historical and mechanistic example of potential cardiac toxicity due to Amphotericin B. The subsequent cases all report reactions to LAmB

Several medications that contain liposomal preparations, including LAmB, have been associated with “Complement activation related pseudoallergy” (CARPA) [15], a process in which complement is activated via both the classical and alternative pathway, giving rise to C3 and C5a anaphylatoxins and subsequent mast cell degranulation. It is possible that our patient experienced such a reaction. This also appears a possible mechanism for some of the previously published cases.

As LAmB is an important agent in the management of severe fungal infections, anaphylaxis to this agent presents a challenging scenario, and understanding the reaction to be either IgE mediated or non-IgE mediated mast cell activation is a potential focus for future research.

To our knowledge, this is the first published occurrence of skin prick and intradermal testing for LAmB allergy. However, whilst SPT (at 1:10 concentration) and IDT was performed at up to 1:100 concentration, maximal non-irritating concentrations are unknown. Clinicians should be aware of the potential for either IgE or non-IgE mediated mast cell reactions in the setting of LAmB, and further research is required into the immunopathogenesis of these reactions.

## Data Availability

All data generated during this study are included in this article.
